# Comprehensive Analysis Reveals Midnolin as a Potential Prognostic, Therapeutic, and Immunological Cancer Biomarker

**DOI:** 10.3390/biomedicines13020276

**Published:** 2025-01-23

**Authors:** Xin-Guo Zhang, Wen-Ting Li, Xin Jin, Chuang Fu, Wen Jiang, Jie Bai, Zhi-Zhou Shi

**Affiliations:** 1 Faculty of Life Science and Technology, Kunming University of Science and Technology, Kunming 650500, China; zhangxinguor@126.com (X.-G.Z.); liwenting2035@163.com (W.-T.L.); 2 Medical School, Kunming University of Science and Technology, Kunming 650500, China; jinxin8833@163.com (X.J.); fuchuang3659@163.com (C.F.); jiebai662001@126.com (J.B.); 3 Department of Thoracic Surgery, The Affiliated Hospital of Kunming University of Science and Technology and First People’s Hospital of Yunnan Province, Kunming 650000, China; coolgzy1314@126.com

**Keywords:** MIDN, prognosis biomarker, immune microenvironment, FTO, breast cancer, gastric cancer

## Abstract

**Background/Objectives**: MIDN (midnolin) is newly discovered method for critically regulating a ubiquitin-independent proteasomal degradation pathway. This study aims to examine the expression, prognostic value, genomic changes, interacting proteins, methylation status, and correlations with the tumor immune microenvironment of MIDN in various cancers. **Methods**: The GTEx, Depmap, GEPIA2, and Kaplan–Meier Plotter databases are applied to evaluate the MIDN level in tumor and normal tissues and the MIDN prognostic value in cancers. The genetic alterations of MIDN in cancers are investigated using the cBioPortal database. The STRING, GeneMANIA, DAVID, and Human Protein Atlas are harnessed to identify and analyze MIDN-interacted proteins. The Sangerbox 3.0 platform (a pan-cancer analysis module) is used to measure the correlations between the MIDN level and the tumor immune microenvironment, stemness, immune cell infiltration, tumor mutational burden, immune checkpoint genes, and RNA modification genes. Immunofluorescence, qRT-PCR, and Western blotting assays were used to evaluate the biological roles of MIDN in breast and gastric cancer cells. **Results**: MIDN expression was dysregulated in many cancers and associated with prognosis in several cancers, such as esophageal cancer. MIDN was mutated in 1.7% of cancers, and deep deletion was the dominant mutation type. NR4A1, PSMC1, and EGR1 were selected as MIDN-interacted proteins, and these four molecules were co-expressed in pancreatic cancer, liver cancer, urothelial cancer, melanoma, and breast cancer. MIDN expression was significantly correlated with the infiltration of CD8+ T cell, CD4+ T cell, B cell, macrophage, neutrophil, and DC both in prostate adenocarcinoma and liver hepatocellular carcinoma. The MIDN level was correlated with several immune checkpoint genes, such as VEGFA, and RNA modification genes such as YTHDF1, YTHDF2, YTHDF3, and YTHDC1 in cancers. Furthermore, in breast cancer cells, the downregulation of MIDN suppressed the colony formation abilities and lessened cell-cycle-associated and stemness-associated genes; in gastric cancer, the knockdown of MIDN diminished the mRNA levels of Nanog and LDHA. Strikingly, silence of MIDN upregulated FTO protein expression in both breast and gastric cancer cells. **Conclusions**: Our findings demonstrate the expression, prognostic value, mutation status, interacting proteins, methylation status, and correlations with the tumor immune microenvironment of MIDN. MIDN will be developed as a potential therapeutic target and a prognosis biomarker.

## 1. Introduction

Cancer ranks as the second leading cause of death, as reported by the WHO [1]. In 2022, new cancer cases and cancer deaths totalled 19,976,499 and 9,743,832, respectively, in the world [2]. With advances in cancer treatment, the cancer death rate continuously declined, but the rate was still astonishingly high [3].

Many drugs have been developed to kill cancer cells by affecting distinct mechanisms. Cemiplimab, an approved anticancer drug targeting PD-1, showed its antitumor effect via rescuing exhausted T cells and activating the immune response [4]. Dysregulation of protein degradation is a hallmark of cancer. For instance, the defects of KEAP1 lose the ability to target EMSY for ubiquitin-mediated degradation and contribute to the development of non-small cell lung cancer [5]. Therefore, targeting protein degradation is a promising approach to cancer therapy, like proteolysis-targeting chimera (PROTAC). Two forms of PROTAC, called ARV-110 and ARV-471, have already entered clinical trials [6].

MIDN (midnolin) is a protein containing a ubiquitin-like domain. In 2013, a study demonstrated that midnolin regulated glucokinase enzyme activity in pancreatic islets and was highly expressed in both the nucleus and cytoplasm [7]. Subsequent studies indicated that midnolin regulated neurite outgrowth and was strongly correlated with Parkinson’s disease because of lower MIDN gene copy numbers [8,9,10]. Another study found that overexpressed midnolin facilitated the progression of hepatocellular carcinoma (HCC), also discovering that the suppression of midnolin disrupted retinoic acid/lipid metabolism in HCC cells and restrained tumor growth [11].

Interestingly, in 2023, Gu et al. discovered that midnolin could promote protein degradation in a ubiquitin-independent and proteasome-dependent manner, which shows great promise for both research and drug development in oncology [12]. Therefore, we systematically analyzed the expression, prognostic value, genomic changes, interacting proteins, and methylation status of MIDN. Furthermore, we analyzed the correlations between MIDN level and immune cell infiltration, stemness, tumor mutational burden (TMB), RNA modification genes, and immune checkpoint genes.

## 2. Materials and Methods

### 2.1. Expression Analysis of MIDN in Normal and Tumor Tissues

The Depmap (https://depmap.org (accessed on 29 October 2023)) and GTEx (https://www.gtexportal.org (accessed on 20 October 2023)) databases are harnessed to investigate the MIDN levels in cancer cell lines and normal tissues. The Sangerbox 3.0 platform (“Pan-cancer analysis” module, http://www.sangerbox.com (accessed on 17 October 2023)) is harnessed to perform a pan-cancer analysis of MIDN expression (TCGA data). The GEPIA2 database (“Pathological Stage Plot” module, http://gepia2.cancer-pku.cn (accessed on 17 October 2023)) is applied to evaluate the correlations between tumor stage and MIDN expression.

### 2.2. Prognosis Analysis of MIDN in Cancers

The Sangerbox 3.0 platform (the “Pan-cancer analysis” module, http://www.sangerbox.com (accessed on 27 October 2023)), GEPIA2 database (“Survival Map” and “Survival Analysis” modules, http://gepia2.cancer-pku.cn (accessed on 27 October 2023)), and Kaplan–Meier Plotter database (http://kmplot.com, pan-cancer RNA-seq (accessed on 24 October 2023)) are used for determining the prognostic value of MIDN in pan-cancer.

### 2.3. Genomic Analysis of MIDN in Cancers

The genetic alteration rate and types, mutation site information, and mutation co-occurrence are analyzed based on the cBioPortal database (https://www.cbioportal.org (accessed on 23 October 2023)) [13].

### 2.4. Analysis of MIDN Interacting Proteins in Cancers

The GeneMANIA (http://genemania.org (accessed on 28 October 2023)) and STRING (https://www.string-db.org (accessed on 22 October 2023)) programs are applied to create PPI (Protein–Protein Interaction) networks for MIDN. GO and KEGG analyses are carried out using the DAVID (https://david.ncifcrf.gov (accessed on 28 October 2023)) database. The Human Protein Atlas (https://www.proteinatlas.org (accessed on 28 October 2023)) database is applied to evaluate the protein levels of selected genes. The subcellular locations of selected genes are analyzed using the Human Protein Atlas (https://www.proteinatlas.org (accessed on 29 October 2023)) database.

### 2.5. Methylation Analysis of MIDN in Cancers

The OncoDB (https://oncodb.org (accessed on 30 October 2023)) database is used to perform the methylation analysis of MIDN in cancers.

### 2.6. Single-Cell State Atlas of MIDN in Cancers

The CancerSEA (http://biocc.hrbmu.edu.cn/CancerSEA/ (accessed on 31 October 2023)) database is applied to estimate the role of MIDN in different biological processes.

### 2.7. Correlation Analyses of MIDN Expression with Tumor Immune Microenvironment, Immune Cell Infiltration, Stemness, Tumor Mutational Burden (TMB), Immune Checkpoint Genes, and RNA Modification Genes in Cancers

The Sangerbox 3.0 platform (http://www.sangerbox.com, “Pan-cancer analysis” module (accessed on 31 October 2023)) is harnessed to perform all these correlation analyses.

### 2.8. Cell Culture

MCF-7 (ATCC, Manassas, VA, USA) and SNU-216 (COBIOER BIOSCIENCE, Nanjing, China) cells are cultured with a DMEM medium and RPMI1640 medium (100 U/mL penicillin, 100 µg/mL streptomycin, and 10% FBS) at 5% CO_2_ and 37 °C, respectively.

Transfection is carried out using lipofectamine 2000 (Thermo, Carlsbad, CA, USA) following the manufacturer’s instructions. Sequences of siRNAs (GenePharma, Shanghai, China) are listed in Table S1.

### 2.9. qRT-PCR Assay

A PowerUp™ SYBR™ Green Master Mix (Thermo, Carlsbad, CA, USA) and HifiScript cDNA Synthesis Kit (Cwbiotech, Beijing, China) are used in the qRT-PCR assay. The sequences of primers (Tsingke, Kunming, China) are listed in Table S2.

### 2.10. Colony Formation Assay

Colony formation ability is measured using the colony formation assay following the previously described [14].

### 2.11. Western Blot Assay

A Western blot procedure is referred to in the previous description [14]. The antibodies’ information includes MIDN antibody (18939-1-AP); FTO antibody (27226-1-AP); ALKBH5 (16837-1-AP) and AIFM2 antibody (20886-1-AP), which are from Proteintech (Wuhan, China); and GAPDH antibody (ab8245, Abcam, Cambridge, UK).

### 2.12. Statistical Analysis

Quantitative data are shown as the mean ± SD and are analyzed by GraphPad Prism 9 software (La Jolla, CA, USA) using a Student’s *t*-test or ANOVA methods. *p* < 0.05 is defined to be significant.

## 3. Results

### 3.1. MIDN Expression in Tumor Tissues, Cancer Cell Lines, and Normal Tissues

**Figure 1:**
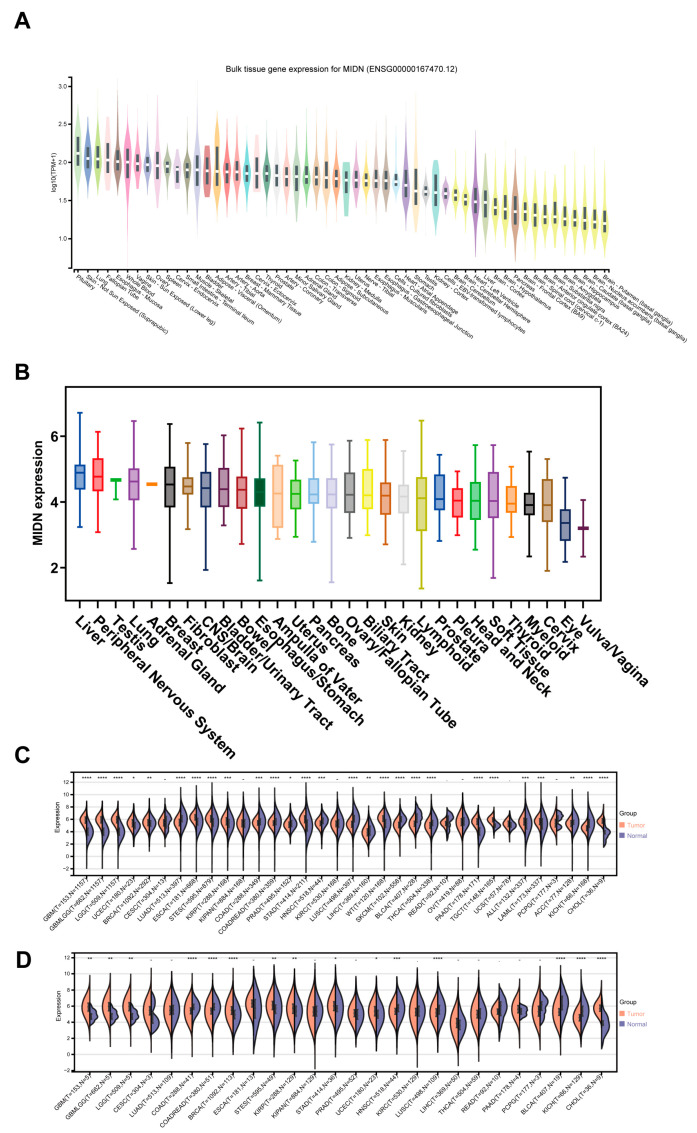
MIDN expression in normal and tumor tissues. (**A**) MIDN mRNA level in normal tissues in the GTEx database. (**B**) MIDN mRNA levels in cancer cell lines in the Depmap database. MIDN expression in tumor tissues and normal tissues in the Sangerbox 3.0 platform (**C**), TCGA, and GTEx data, and (**D**) TCGA data. -, not significant; *, *p* < 0.05; **, *p* < 0.01; ***, *p* < 0.001; ****, *p* < 0.0001.

First, using the GTEx database, we evaluated the MIDN mRNA levels in normal tissues and found that the top five tissues with the highest MIDN expression were pituitary, non-sun-exposed skin (suprapubic), lung, fallopian tube, and esophagus-mucosa, whereas the brain tissues, such as putamen (basal ganglia) and nucleus accumbens (basal ganglia), had the lowest level (Figure 1A). Analysis of the Depmap database showed that MIDN was highly detected in liver cancer as well as peripheral nervous system cancer, whereas it was lowly expressed in vulva/vagina cancer, eye cancer, and so on (Figure 1B). Pan-cancer analysis (TCGA data) using the Sangerbox 3.0 platform suggested that MIDN was overexpressed in cholangiocarcinoma (CHOL), uterine corpus endometrial carcinoma (UCEC), stomach adenocarcinoma (STAD), kidney renal papillary cell carcinoma (KIRP), stomach and esophageal carcinoma (STES), breast invasive carcinoma (BRCA), brain lower grade glioma (LGG), glioblastoma multiforme and brain lower grade glioma (GBMLGG), and glioblastoma multiforme (GBM), and declined in kidney chromophobe (KICH), bladder urothelial carcinoma (BLCA), lung squamous cell carcinoma (LUSC), head and neck squamous cell carcinoma (HNSC), colon and rectum carcinoma (COADREAD), and colon carcinoma (COAD, Figure 1C,D).

### 3.2. Correlation Analysis of MIDN Level with Cancer Stages

**Figure 2:**
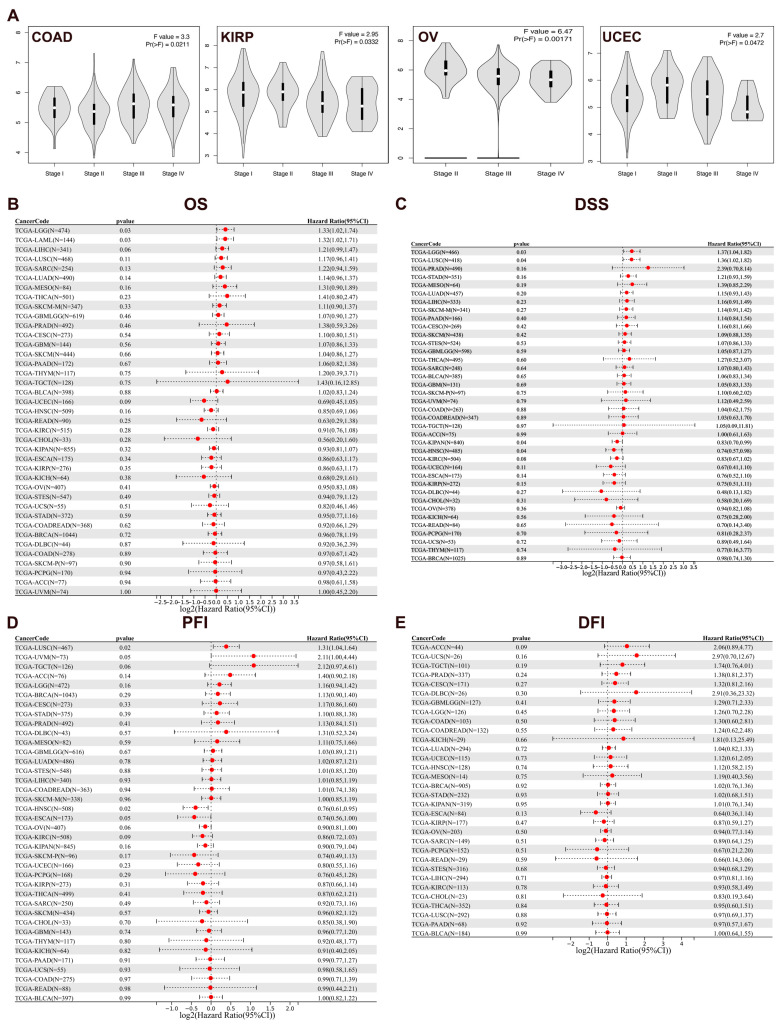
Correlation analysis of MIDN expression with cancer stages and prognosis. (**A**) The correlation (GEPIA2 database) between MIDN expression and stages of COAD, KIRP, OV, and UCEC. The correlation (Sangerbox 3.0 platform) between MIDN expression and OS (**B**), DSS (**C**), PFI (**D**), and DFI (**E**) in cancers.

The “Pathological Stage Plot” module (GEPIA2) is applied to analyze the correlations between MIDN expression and stages of cancers. The MIDN mRNA level was markedly linked with stages of COAD, KIRP, ovarian serous cystadenocarcinoma (OV), and UCEC (Figure 2A, *p* < 0.05).

### 3.3. Prognosis Value of MIDN Expression in Cancers

Then, we evaluated the prognostic value of MIDN mRNA levels in pan-cancer using the Sangerbox 3.0 platform. The high expression level of MIDN was correlated with unfavorable overall survival (OS) in patients with LGG and acute myeloid leukemia (LAML), with poor disease-specific survival (DSS) in patients of LGG and LUSC, and with a shortened progression-free interval (PFI) in LUSC patients (Figure 2B–D). However, there is no association between MIDN expression and disease-free interval (DFI) of cancer patients (Figure 2E).

**Figure 3:**
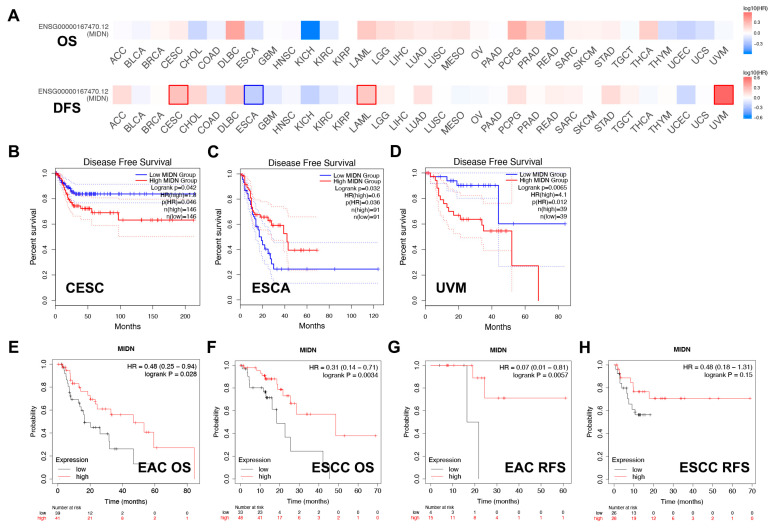
Correlation analysis of MIDN expression with cancer prognosis. (**A**) Survival map of MIDN expression in pan-cancer (GEPIA2 database). The correlation (GEPIA2 database) between MIDN expression and DFS in CESC (**B**), ESCA (**C**), and UVM (**D**), and the dotted line indicates the 95% confidence interval. The correlation (Kaplan–Meier Plotter database) between MIDN expression and OS in EAC (**E**) and ESCC (**F**), and RFS in EAC (**G**) and ESCC (**H**).

The prognosis value of MIDN expression in pan-cancer is also analyzed using the GEPIA2 and Kaplan–Meier Plotter databases. In the GEPIA2 database, high MIDN expression was linked with shortened disease-free survival (DFS) in patients with uveal melanoma (UVM) and cervical squamous cell carcinoma and endocervical adenocarcinoma (CESC), as well as with prolonged DFS in patients of esophageal carcinoma (ESCA, Figure 3A–D). In the Kaplan–Meier Plotter database, MIDN expression was also negatively associated with the OS and relapse-free survival (RFS) in esophageal squamous cell carcinoma (ESCC) and esophageal adenocarcinoma (EAC, Figure 3E–H).

### 3.4. Genomic Changes in MIDN in Cancers

**Figure 4:**
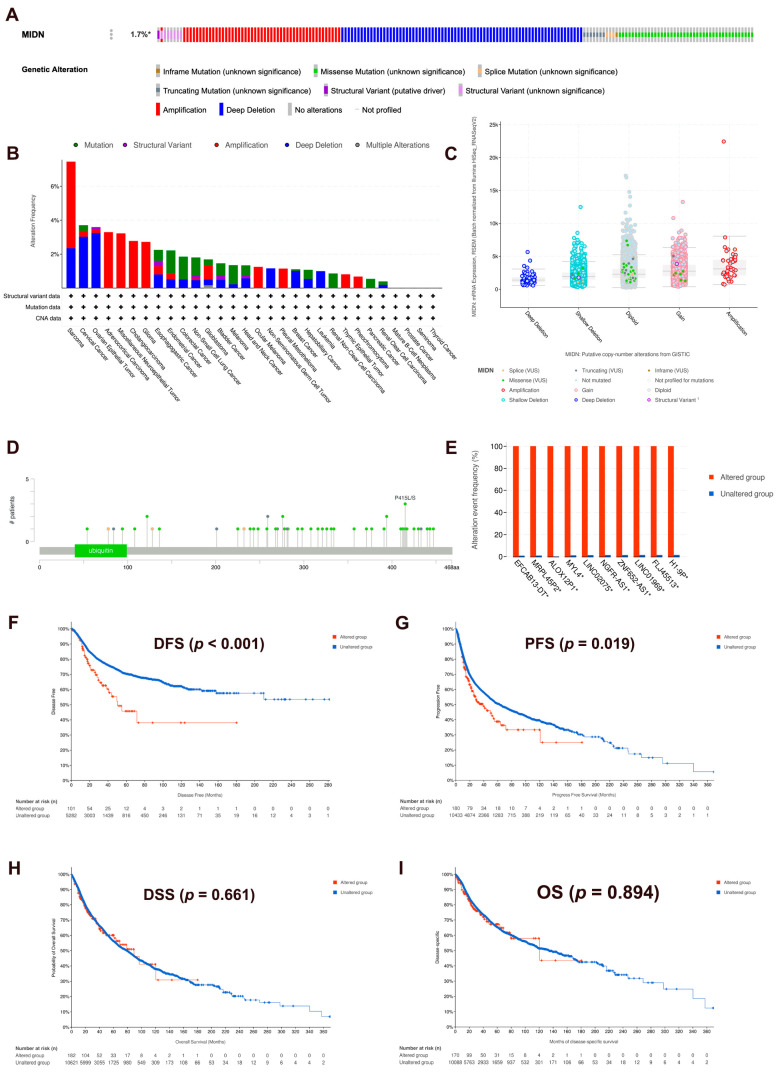
Genomic changes in MIDN in cancers. (**A**) Summary of MIDN genetic alterations, and the asterisk indicates “not all samples are profiled”. (**B**) MIDN genetic alterations in TCGA pan-cancer data. (**C**) Copy number changes in MIDN in TCGA pan-cancer data. (**D**) Mutation information of MIDN genes. (**E**) The co-occurrence of gene alteration in the MIDN-altered group and unaltered group. Correlations of MIDN alterations with DFS (**F**), PFS (**G**), DSS (**H**), and OS (**I**) in TCGA pan-cancer data.

Based on the cBioportal database, we analyzed the genomic changes in MIDN in cancers. Figure 4A shows that the MIDN genetic mutation frequency is 1.7% and that the main types are deep deletion, amplification, and missense mutation. The top three mutation frequencies were detected in sarcoma, cervical cancer, and ovarian epithelial cancer (Figure 4B). GISTIC analysis results showed that MIDN genomic changes, including amplification, gain, diploid, shallow deletion, and deep deletion, affected its expression (Figure 4C). Figure 4D displayed the sites and numbers of MIDN gene mutations in cancers, and P415L/S was the most common mutation site. After genetic mutation co-occurrence analysis, EFCAB13-DT, MRPL45P2, ALOX12P1, MYL4, LINC02075, NGFR-AS1, ZNF652-AS1, LINC01969, FLJ45513, and H1-9P were found to be more frequently changed in the MIDN-altered group than the MIDN-unaltered group (Figure 4E). Furthermore, we also found that MIDN-altered cancer patients had unfavorable DFS and progression-free survival (PFS), with no correlation between DSS and OS (Figure 4F–I).

### 3.5. Interacting Proteins of MIDN in Cancers

**Figure 5:**
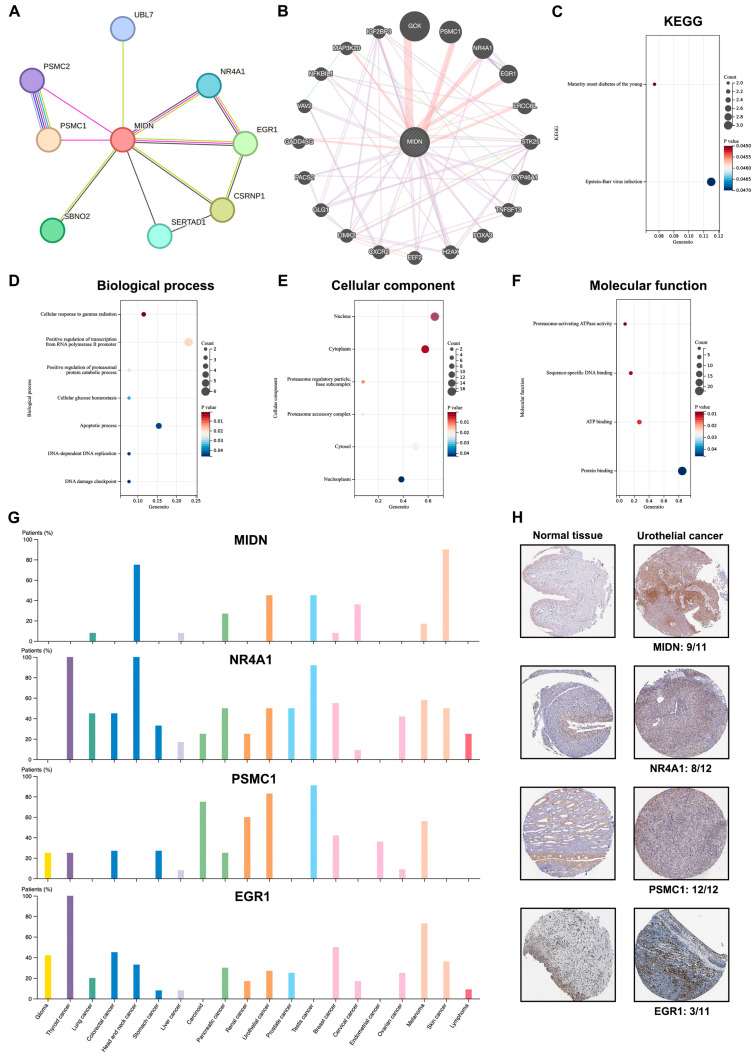
Interacting proteins of MIDN in cancers. The PPI networks were created by the STRING (**A**) and GeneMANIA (**B**) programs. KEGG (**C**), biological process (**D**), cellular component (**E**), and molecular function (**F**) enrichment analyses were carried out by the DAVID software (https://david.ncifcrf.gov (accessed on 28 October 2023)). (**G**) The protein levels of MIDN, NR4A1, PSMC1, and EGR1 in cancers were evaluated using the Human Protein Atlas database. (**H**) The immunohistochemistry staining of MIDN, NR4A1, PSMC1, and EGR1 in urothelial cancer (Human Protein Atlas database).

The STRING and GeneMANIA programs are applied to create PPI networks for MIDN (Figure 5A,B), and NR4A1, PSMC1, and EGR1 were selected as MIDN-interacted proteins by two programs. All MIDN-interacted proteins together with MIDN were utilized to carry out KEGG and GO enrichment analyses. KEGG enrichment analysis indicated that MIDN-interacted proteins were associated with maturity onset diabetes in young patients and the Epstein–Barr virus infection (Figure 5C). Biological process enrichment analysis suggested that MIDN-interacted proteins were correlated with cellular response to gamma radiation, positive regulation of transcription from RNA polymerase II promoter, and so on (Figure 5D). Cellular component enrichment analysis indicated that MIDN-interacted proteins were correlated with nucleus, cytoplasm, and so on (Figure 5E). Molecular function enrichment analysis suggested that MIDN-interacted proteins were linked with proteasome-activating ATPase activity, sequence-specific DNA binding, and so on (Figure 5F).

**Figure 6:**
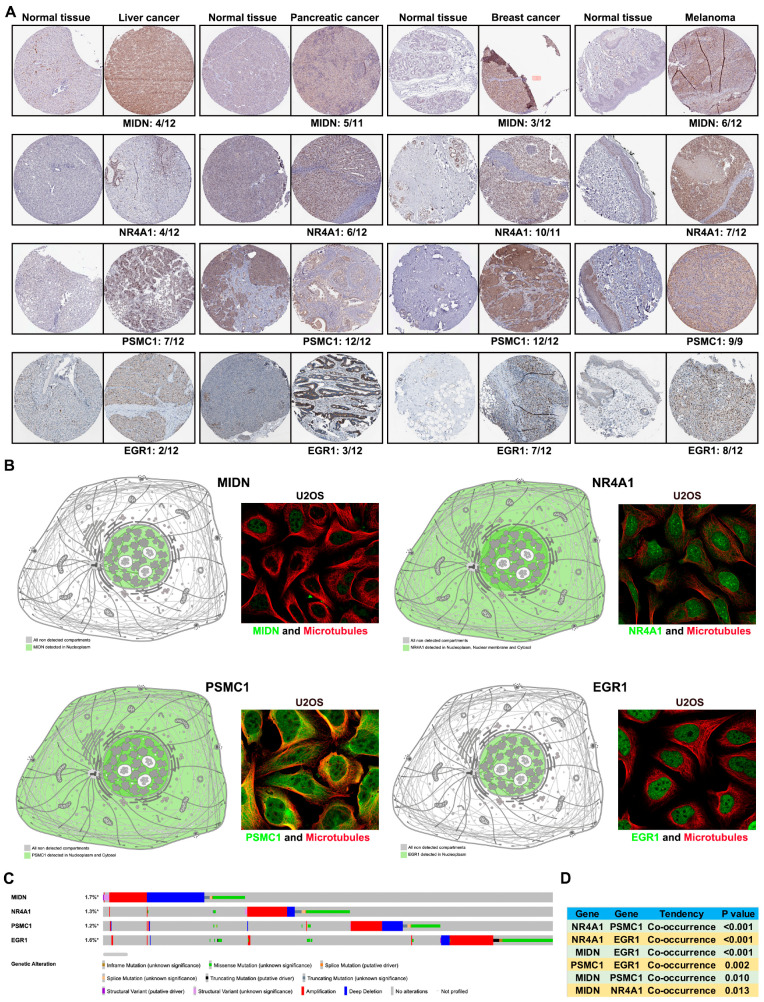
Interacting proteins of MIDN in cancers. (**A**) Immunohistochemistry staining of MIDN, NR4A1, PSMC1, and EGR1 in liver cancer, pancreatic cancer, breast cancer, and melanoma (Human Protein Atlas database). (**B**) Subcellular locations of MIDN, NR4A1, PSMC1, and EGR1 in HPA database. (**C**) Summary of genetic alterations of MIDN, NR4A1, PSMC1, and EGR1, and the asterisk indicates “not all samples are profiled”. (**D**) The co-occurrence analysis among MIDN, NR4A1, PSMC1, and EGR1 in TCGA pan-cancer data.

Based on the Human Protein Atlas (HPA) database, we found that MIDN, NR4A1, PSMC1, and EGR1 were all positively expressed in liver cancer, pancreatic cancer, urothelial cancer, breast cancer, and melanoma (Figure 5G). In urothelial cancer, the frequencies of MIDN, NR4A1, PSMC1, and EGR1 positive expression were 9/11, 8/12, 12/12, and 3/11, respectively (Figure 5H). Figure 6A showed the frequencies of MIDN, NR4A1, PSMC1, and EGR1 positive expression in liver cancer, pancreatic cancer, breast cancer, and melanoma, respectively (Figure 6A). We further analyzed the subcellular locations of MIDN, NR4A1, PSMC1, and EGR1 using the HPA database. Interestingly, all four of these genes were at least located in nucleoplasm (Figure 6B). After analyzing the cBioportal database, co-occurrence tendencies (mutation) were found between NR4A1 and PSMC1, between NR4A1 and EGR1, between MIDN and EGR1, and so on (Figure 6C,D).

### 3.6. Methylation Status of MIDN in Cancers

**Figure 7:**
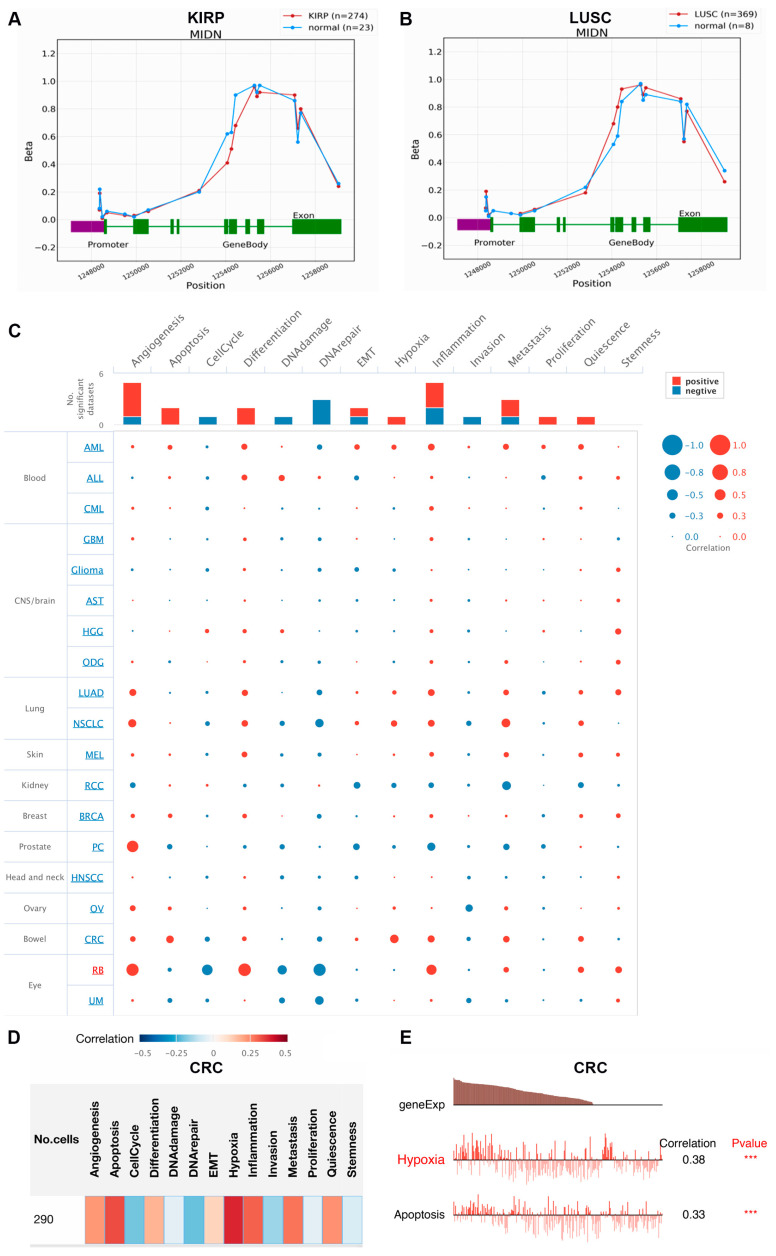
Methylation status and single-cell analysis of MIDN in cancers. The methylation status of the MIDN gene in KIRP (**A**) and LUSC (**B**). (**C**–**E**) The roles of MIDN in different biological processes were analyzed by the CancerSEA database, and *** indicates “*p* < 0.001”.

The OncoDB database is used to perform the methylation analysis of MIDN in cancers. Compared with normal tissues, MIDN was hypomethylated in KIRP and was hypermethylated in LUSC (Figure 7A,B).

### 3.7. Single-Cell Analysis of MIDN in Cancers

The role of MIDN in different biological processes is estimated using the CancerSEA database. Figure 7C showed that MIDN expression was associated with angiogenesis, inflammation, and metastasis in 19 types of cancers. In colorectal cancer in particular, MIDN expression was linked with hypoxia as well as apoptosis (correlation coefficients: 0.38 and 0.33; Figure 7D,E).

### 3.8. Correlation Analysis of the Tumor Immune Microenvironment with MIDN Levels in Cancers

**Figure 8:**
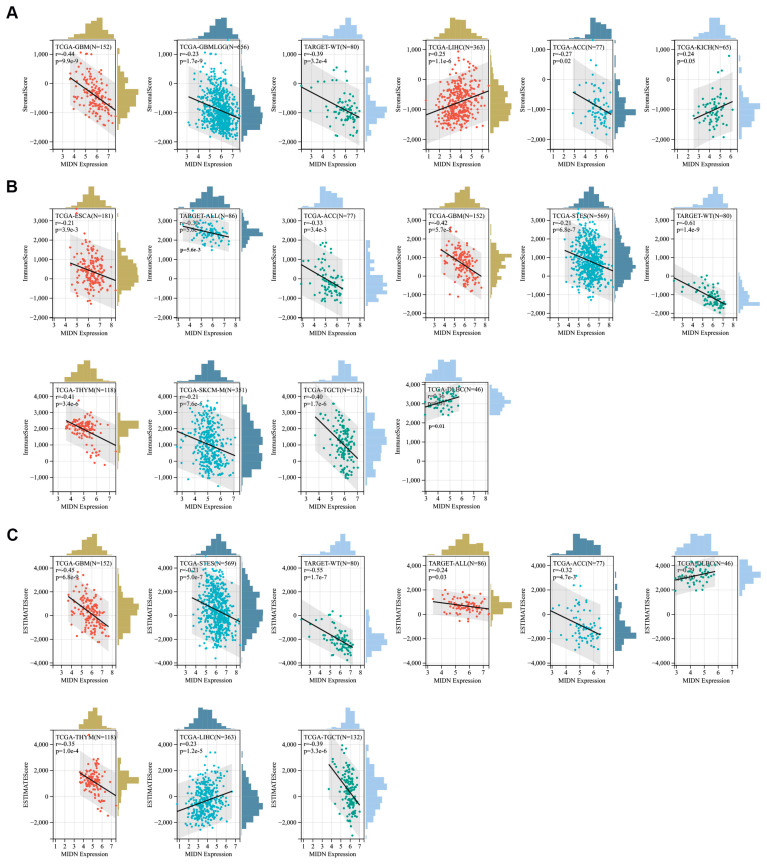
Correlation analysis of MIDN expression with the tumor immune microenvironment in cancers. The significant correlations between MIDN expression and immune infiltration (**A**), StromalScore; (**B**), ImmuneScore; (**C**), ESTIMATEScore.

Using the estimate module of the Sangerbox 3.0 platform, we revealed that MIDN level and the stromal score were positively correlated in liver hepatocellular carcinoma (LIHC) and KICH and negatively correlated in GBM, GBMLGG, adrenocortical carcinoma (ACC), and high-risk Wilms tumor (WT, Figure 8A). MIDN level and the immune score were positively correlated in lymphoid neoplasm diffuse large B cell lymphoma (DLBC), and negatively associated in ESCA, acute lymphoblastic leukemia (ALL), ACC, GBM, STES, WT, testicular germ cell tumors (TGCTs), skin cutaneous melanoma (SKCM), and thymoma (THYM, Figure 8B). MIDN expression and the estimate score were positively linked in DLBC and LIHC and negatively linked in GBM, STES, WT, ALL, ACC, THYM, and TGCTs (Figure 8C).

### 3.9. Correlation Analysis of the Immune Cell Infiltration, Stemness, and Tumor Mutational Burden with MIDN Expression in Cancers

**Figure 9:**
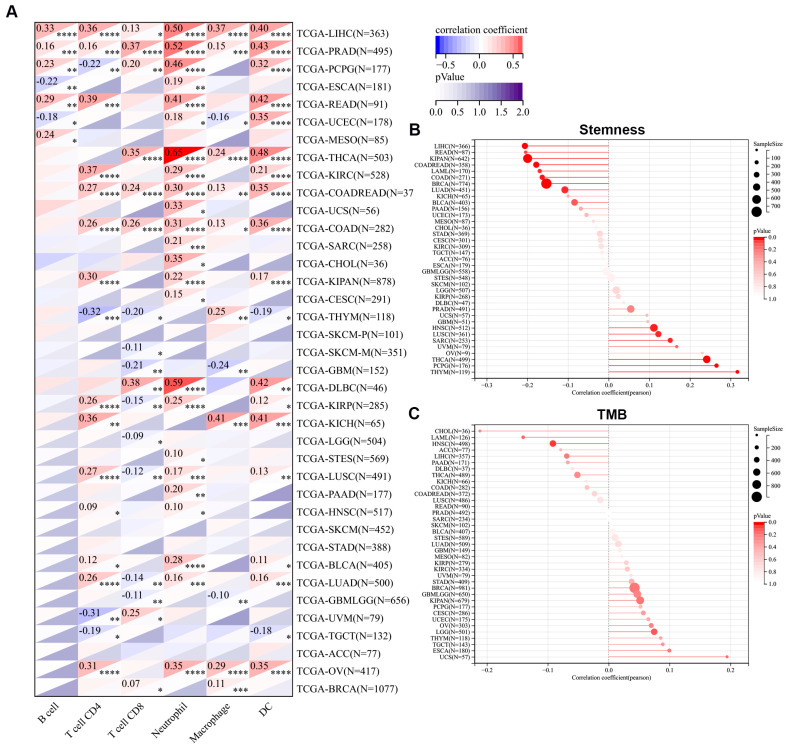
Correlation analysis of MIDN expression with the immune cell infiltration, stemness, and tumor mutational burden in cancers. (**A**) Correlations between MIDN expression and the immune cell infiltration in cancers. *, *p* < 0.05; **, *p* < 0.01; ***, *p* < 0.001; ****, *p* < 0.0001. (**B**) Correlations between MIDN expression and stemness in cancers. (**C**) Correlations between MIDN expression and tumor mutational burden in cancers.

Based on the Timer module of the Sangerbox 3.0 platform, the correlations between immune cell infiltration and MIDN expression were analyzed. Strikingly, the MIDN level was remarkably associated with infiltration of DC, macrophage, neutrophil, CD8+ T cell, CD4+ T cell, and B cell both in LIHC and PRAD. Importantly, the highest correlation was detected between the MIDN expression and infiltration of neutrophils in thyroid carcinoma (THCA, Figure 9A).

The correlation of MIDN level with stemness in cancers was also evaluated using the stemness module of the Sangerbox 3.0 platform. The MIDN level was positively linked with the stemness in THYM, pheochromocytoma and paraganglioma (PCPG), THCA, and so on, and negatively associated with the stemness in rectum adenocarcinoma (READ), LIHC, and so on (Figure 9B).

The correlations of MIDN level with TMB were analyzed using the tumor heterogeneity module of the Sangerbox 3.0 platform. The MIDN level was positively linked with the TMB of uterine carcinosarcoma (UCS) and negatively linked with the TMB of CHOL (Figure 9C).

### 3.10. Correlation Analysis of the Immune Checkpoint Genes with MIDN in Cancers

**Figure 10:**
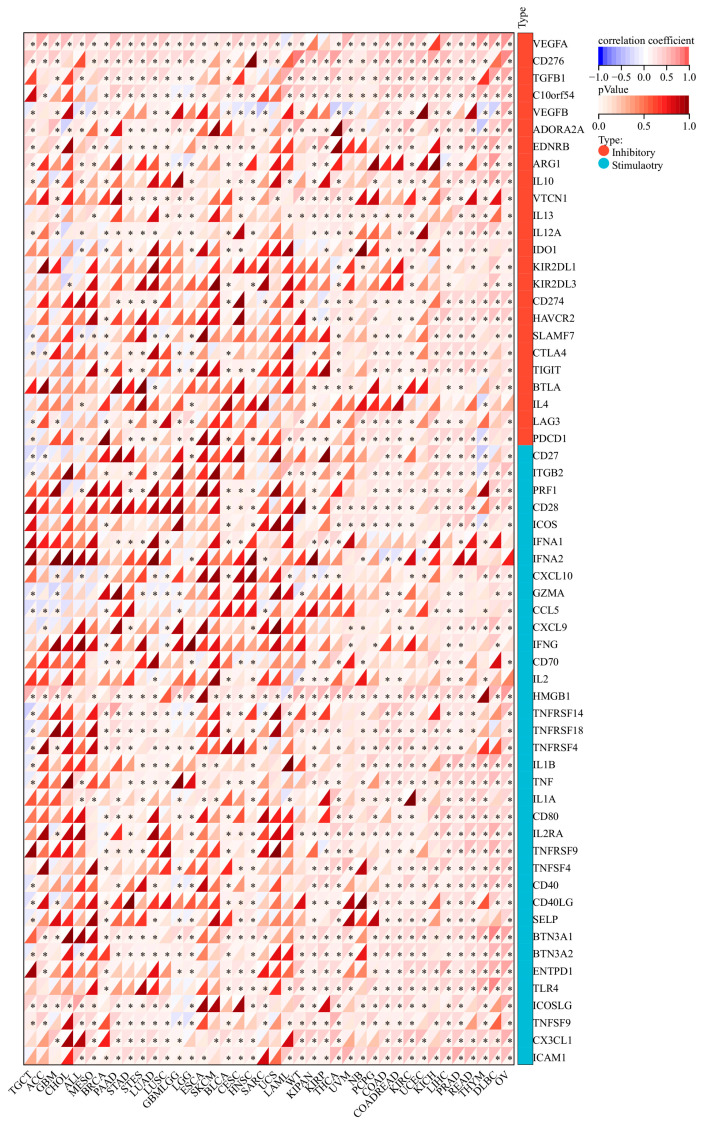
Correlation analysis of MIDN expression with the immune checkpoint genes in cancers. *, *p* < 0.05.

Using the Immune checkpoint module of the Sangerbox 3.0 platform, we discovered that MIDN expression was linked with the expression of many immune checkpoint genes in pan-cancer. For example, the MIDN level was positively linked with the VEGFA level in TGCTs, ACC, GBM, and so on (Figure 10); the MIDN level was positively associated with HMGB1 levels in TGCTs, ACC, GBM, and so on (Figure 10).

### 3.11. Correlation Analysis of the RNA Modification Genes and MIDN in Cancers

**Figure 11:**
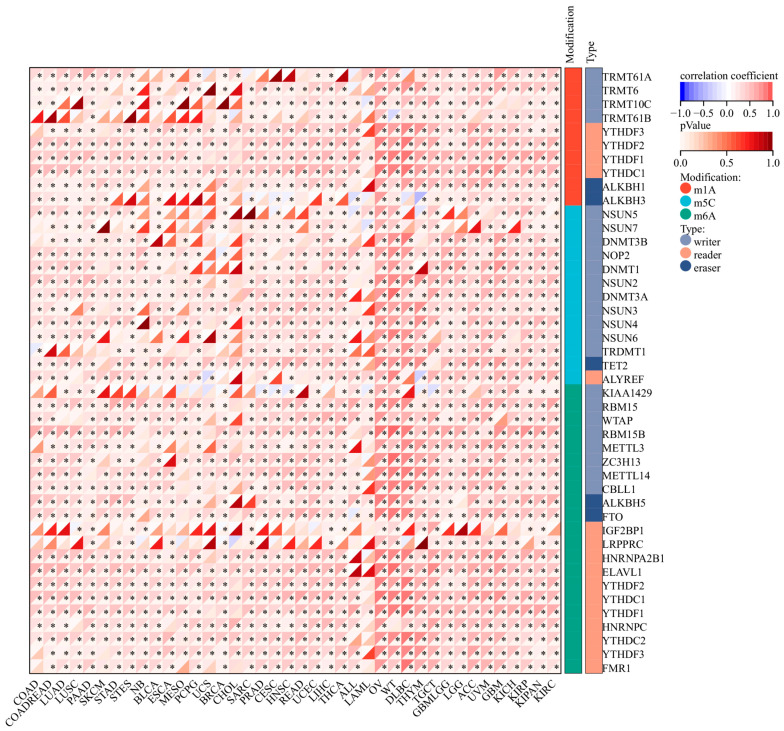
Correlation analysis of MIDN expression with the RNA modification genes in cancers. *, *p* < 0.05.

Using the RNA modification module of the Sangerbox 3.0 platform, we discovered that MIDN expression was linked with levels of many RNA modification molecules in pan-cancer. For example, MIDN expression was found to be positively linked with all m6A-, m5C-, and m1A-associated molecules in ovarian serous cystadenocarcinoma (OV, Figure 11). Positive correlations between the expression of YTHDF3, YTHDF2, YTHDF1, and YTHDC1 and MIDN levels were detected in nearly all cancers (Figure 11).

### 3.12. The Knockdown of MIDN Suppresses Colony Formation and Declines the Expression of Cell Cycle-Associated and Stemness-Associated Genes in Breast Cancer

**Figure 12:**
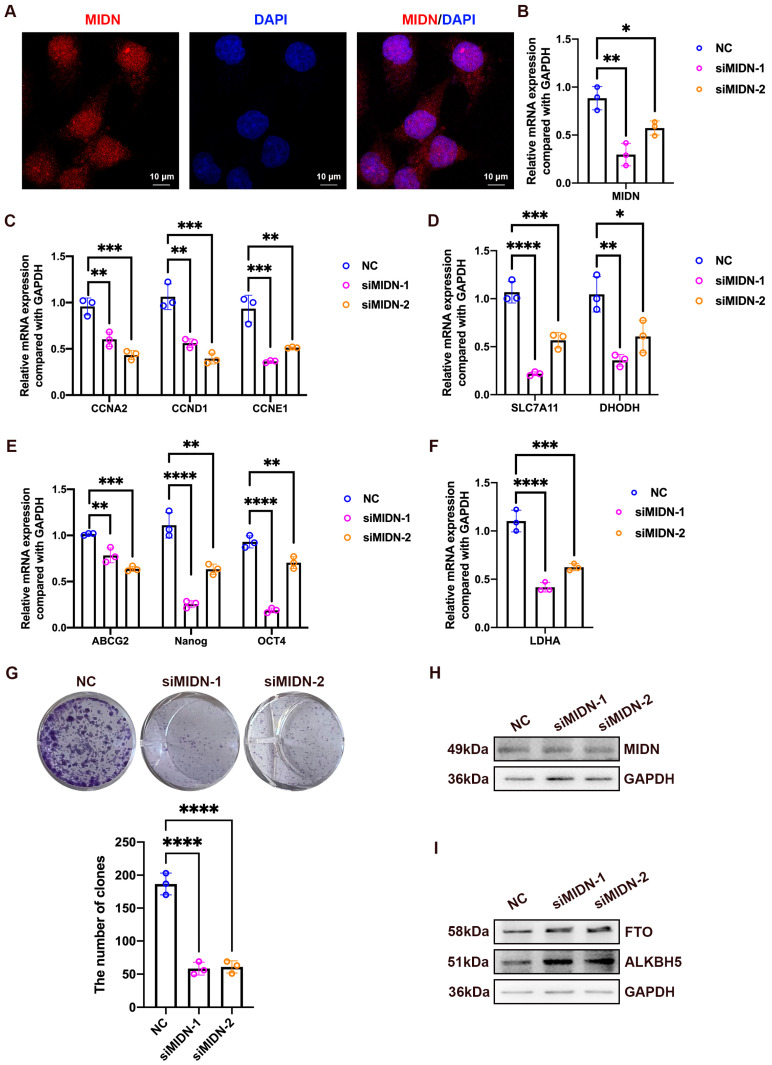
The knockdown of MIDN suppresses colony formation and declines the expression of cell cycle-associated and stemness-associated genes in breast cancer. (**A**) The immunofluorescence staining of MIDN in MCF-7 breast cancer cells. (**B**–**F**) qRT-PCR analysis of interested genes. (**G**) Colony formation ability after MIDN knockdown. (**H**,**I**) The protein levels of MIDN, FTO, and ALKBH5 were detected using a Western blotting assay. *, *p* < 0.05; **, *p* < 0.01; ***, *p* < 0.001; ****, *p* < 0.0001.

The above results have indicated that MIDN was overexpressed in breast cancer tissues (Figure 1C,D), which observed the highest incidence of all cancers [2]; therefore, we further investigated the function of MIDN in the breast cancer cells. The immunofluorescence assay showed that MIDN was expressed both in the cytoplasm and nucleus of MCF-7 cells (Figure 12A). The knockdown of MIDN diminished the expression of cell cycle-associated genes, including CCNA2, CCND1, and CCNE1, ferroptosis-associated genes, including SLC7A11 and DHODH, stemness-associated genes, including ABCG2, Nanog, and OCT4, and a glycolysis-associated gene, LDHA (Figure 12B–F). The silence of MIDN significantly attenuated the colony formation ability of MCF-7 cells (Figure 12G). Importantly, the downregulation of MIDN upregulated m6A demethylases FTO and ALKBH5 in MCF-7 cells (Figure 12H,I).

### 3.13. Knockdown of MIDN Upregulates FTO in Gastric Cancer

**Figure 13:**
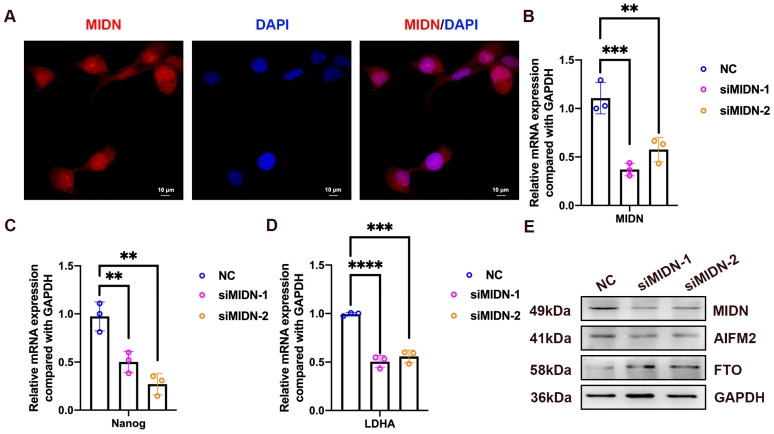
The knockdown of MIDN upregulates FTO in gastric cancer. (**A**) The immunofluorescence staining of MIDN in SNU-216 gastric cancer cells. (**B**–**D**) qRT-PCR analysis of interested genes. (**E**) Protein levels of MIDN, AIFM2, and FTO were detected using a Western blotting assay. **, *p* < 0.01; ***, *p* < 0.001; ****, *p* < 0.0001.

The above results have shown that MIDN was overexpressed in gastric cancer tissues (Figure 1C,D), ranking sixth and seventh in morbidity and mortality in cancers [2]; therefore, we further investigated the function of MIDN in the gastric cancer cells. The immunofluorescence assay showed that MIDN was expressed both in the cytoplasm and nucleus of SNU-216 cells (Figure 13A). A knockdown of MIDN reduced a stemness-associated gene Nanog and a glycolysis-associated gene LDHA (Figure 13B–D). Strikingly, the silence of MIDN downregulated the ferroptosis-associated molecule AIFM2 and upregulated the m6A demethylase FTO (Figure 13E).

Our study uniquely identifies MIDN’s overexpression in rare cancers, such as cholangiocarcinoma, and highlights its prognostic value across diverse cancer types, providing novel insights into its potential as a therapeutic target.

## 4. Discussion

MIDN, also known as midnolin, has recently been found to critically regulate a ubiquitin-independent proteasomal protein degradation pathway [12]. MIDN contains three domains: the α Helix-C domain connects midnolin to the proteasome, the Catch domain directly binds with substrates, and the ubiquitin-like (Ubl) domain promotes substrate degradation [12]. A previous study identified a MIDN homolog Stuxnet in Drosophila and revealed that Stuxnet promoted the protein degradation of Polycomb in a ubiquitin-independent way [15], indicating that the midnolin-proteasome pathway is a conserved protein degradation mechanism. Another study reported that, in the lymphocytes, MIDN was dominantly expressed and enhanced proteasome activity via its C-terminal and ubiquitin-like domains. Additionally, blocking MIDN inhibited B cell malignancies [16]. Importantly, our study found that MIDN expression was positively correlated with B cell infiltration in LIHC, indicating that MIDN might affect the B cell function in LIHC.

MIDN is associated with Parkinson’s disease (PD) progression [10,17]. Notably, 10.5% of PD patients had a lower MIDN gene copy number, whereas healthy controls had no copy number variation. The knockout and knockdown of MIDN significantly downregulated parkin via impeding ATF4- and CRE-dependent transcription activity [9]. The copy number loss of MIDN showed a strong correlation with PD development in the Japanese and British population [8].

Analysis of the TCGA data using the Sangerbox 3.0 platform showed that MIDN was slightly decreased in LIHC compared with normal tissues (including the TCGA and GTEx data), and, when compared with normal tissues (only TCGA data), the expression levels in LIHC and normal tissues were not different. Importantly, a study reported that blocking MIDN suppressed the proliferation, colony formation, and tumor growth of liver cancer cells and mechanically disrupted retinoic acid and lipid metabolisms [11], indicating that MIDN exhibited oncogenic roles in liver cancer. Therefore, to elaborate the functional role of MIDN in liver cancer, the protein expression level and, especially, the nuclear expression of MIDN need to be analyzed. AIPC (androgen-independent prostate cancer) cells could release exosomes and transfer miR-222-3p to ADPC (androgen-dependent prostate cancer) cells, subsequently promoting the transformation to AIPC-like cells mechanically through regulating the MIDN/mTOR pathway [18]. Nevertheless, the roles of MIDN in cancer are largely unclear. Hence, we systematically analyzed the expression, prognostic value, genomic changes, interacting proteins, and methylation status of MIDN. Furthermore, we performed the correlation analyses of MIDN levels with stemness, tumor mutational burden (TMB), immune checkpoint genes, immune cell infiltration, and RNA modification genes in cancers. All the findings will benefit us in understanding the roles of MIDN in tumorigenesis.

Both the GEPIA2 and Kaplan–Meier Plotter databases revealed that high expression of MIDN was associated with favorable outcome of esophageal cancer patients, indicative of the robustness of results across different databases. Interestingly, the MIDN-interacted proteins were enriched in the pathway of Epstein–Barr virus (EBV) infection. EBV infection is involved in the progression of several cancers. In gastric cancer, EBV infection promotes the upregulation and secretion of OLFM4 by potentiating the cGAS-STING pathway, eventually augmenting the cancer progression [19]. Whether and how MIDN regulates the EBV-related cancer progression still requires exploration.

Strikingly, both STRING and GeneMANIA programs selected NR4A1, PSMC1, and EGR1 as MIDN-interacted proteins. The interaction between MIDN and EGR1 has been confirmed. Gu et al. reported that, as immediate-early-genes (IEGs), NR4A1 and EGR1 were the direct targets of MIDN for degradation [12]. Another study further revealed that MIDN interacted with EGR1 and improved its binding ability to the *Nefl* promoter, transcriptionally activating *Nefl* expression in PC12 cells [20]. PSMC1 is a proteasome component, and its potential interaction with MIDN might indicate direct binding between MIDN and proteasome. NR4A1 is a key regulator of T cell dysfunction, mechanically inhibiting AP-1 function and effector-gene expression and promoting H3K27ac and tolerance-related gene expression [21]. Overexpression of NR4A1 improved the carcinogenesis of breast cancer cells, whereas its loss had the opposite effect. Mechanically, NR4A1 bound with the 3′UTR and gene body of IEGs, generating accessible chromatin domains that were detected in about half of breast and other cancers [22]. Whether the oncogenic roles of NR4A1 are regulated by MIDN still requires investigation.

Our study further examined the biological functions of MIDN in breast and gastric cancers in which MIDN was overexpressed. Results showed that the knockdown of MIDN suppressed the colony formation of breast cancer cells and upregulated the demethylase FTO both in breast and gastric cancer cells. FTO plays dual roles in breast cancer progression. On the one hand, FTO was highly expressed in breast cancer and linked with unfavorable survival, advanced TNM stage, and lymph node metastasis [23,24]. Functional studies revealed that FTO promoted breast cancer progression by affecting miR-181b-3p/ARL5B signaling and directly regulating the m6A methylation of 3′UTR of BNIP3 mRNA [23,24]. On the other hand, overexpression of the demethylase FTO restrained the proliferation, invasion, and migration in triple-negative breast cancer cells via affecting the miR-17-5p/ZBTB4 axis [25].

FTO also plays dual roles in gastric cancer development. FTO was overexpressed in gastric cancer tissues and remarkably associated with liver metastasis and poor prognoses [26,27]. FTO enhanced the proliferation and metastasis of gastric cancer cells via potentiating caveolin-1 mRNA degradation [23]. Another study reported that FTO augmented gastric cancer metastasis by upregulating ITGB1 via decreasing its m6A levels [28]. The silence of FTO alleviated the resistance of gastric cancer cells to cisplatin via blocking ULK1-related autophagy [29]. On the contrary, a higher expression of FTO was correlated with favorable outcomes for Epstein–Barr virus-associated gastric cancer (EBVaGC) patients, and FTO suppressed metastasis and aggressiveness via decreasing FOS in EBVaGC cells [30]. Significantly, our study revealed that MIDN was highly expressed in gastric and breast cancers, and the knockdown of MIDN upregulated FTO protein levels both in SNU-216 and MCF-7 cells. The target genes of FTO determine its promotive or suppressive effects on tumor progression; therefore, how the MIDN/FTO axis affects tumor progression urgently needs to be investigated in the future.

Our results indicated that the knockdown of MIDN downregulated AIFM2 in gastric cancer. AIFM2, also known as FSP1, is an anti-ferroptosis molecule [31]. FSP1 led to the resistance of non-small cell lung carcinoma cells (a KEAP1 mutant) to ferroptosis [32]. Blocking AIFM2 strongly sensitized tumor cells to ferroptosis and could be developed as a promising antitumor method [33]. AIFM2 was overexpressed in gastric cancer tissues [34]; however, its roles in gastric cancer progression are unclear.

However, while our findings have deepened our knowledge of the roles of MIDN in tumorigenesis, there are still some unresolved problems. First, the oncogenic and tumor-suppressive proteins targeted by MIDN need to be identified. Second, whether or not targeting MIDN-mediated protein degradation can be developed into a new therapeutic method still requires investigation. Third, the biological function of cytoplasmic MIDN requires further research. Fourth, our findings revealed the oncogenic roles of MIDN in breast and gastric cancers; however, its roles in other types of cancers still need to be investigated.

## 5. Conclusions

In summary, our findings indicated that MIDN was overexpressed in many cancers such as gastric cancer and breast cancer, and under-expressed in a lot of cancers, such as COAD. MIDN interacted with NR4A1, EGR1, and PSMC1, and it was especially co-expressed in liver cancer, pancreatic cancer, urothelial cancer, breast cancer and melanoma. The knockdown of MIDN suppressed the colony formation of breast cancer cells and upregulated FTO both in breast and gastric cancer.

## Data Availability

All data can be found in the GTEx (https://www.gtexportal.org), Depmap (https://depmap.org), GEPIA2 (http://gepia2.cancer-pku.cn), Kaplan–Meier Plotter (http://kmplot.com), cBioPortal (https://www.cbioportal.org), GeneMANIA (http://genemania.org), STRING (https://www.string-db.org), DAVID (https://david.ncifcrf.gov), OncoDB (https://oncodb.org), CancerSEA (http://biocc.hrbmu.edu.cn/CancerSEA/), and Sangerbox 3.0 platform (http://www.sangerbox.com). All data are publicly available, accessed on 29 December 2024.

## Supplementary Materials

The following supporting information can be downloaded at: https://www.mdpi.com/article/10.3390/biomedicines13020276/s1, Table S1: Sequence information of siRNAs; Table S2: Sequence information of primers.

## Abbreviations

ACC	adrenocortical carcinoma
ADPC	androgen-dependent prostate cancer
AIPC	androgen-independent prostate cancer
ALL	acute lymphoblastic leukemia
BLCA	bladder urothelial carcinoma
BRCA	breast invasive carcinoma
CESC	cervical squamous cell carcinoma
CHOL	cholangiocarcinoma
COAD	colon carcinoma
COADREAD	colon and rectum carcinoma
DFI	disease-free interval
DFS	disease-free survival
DLBC	diffuse large B cell lymphoma
DSS	disease-specific survival
EAC	esophageal adenocarcinoma
EBVaGC	Epstein–Barr virus-associated gastric cancer
ESCA	esophageal carcinoma
ESCC	esophageal squamous cell carcinoma
GBM	glioblastoma multiforme
GBMLGG	glioblastoma multiforme and brain lower grade glioma
HCC	hepatocellular carcinoma
HNSC	head and neck squamous cell carcinoma
HPA	Human Protein Atlas
IEGs	immediate-early-genes
KICH	kidney chromophobe
KIRP	kidney renal papillary cell carcinoma
LAML	LGG and acute myeloid leukemia
LGG	lower grade glioma
LIHC	liver hepatocellular carcinoma
LUSC	lung squamous cell carcinoma
MIDN	midnolin
OS	overall survival
OV	ovarian serous cystadenocarcinoma
PCPG	pheochromocytoma and paraganglioma
PD	Parkinson’s disease
PFI	progression-free interval
PFS	progression-free survival
PROTAC	proteolysis-targeting protein
READ	rectum adenocarcinoma
RFS	relapse-free survival
SKCM	skin cutaneous melanoma
STAD	stomach adenocarcinoma
STES	stomach and esophageal carcinoma
TGCTs	testicular germ cell tumors
THYM	thymoma
TMB	tumor mutational burden
TMB	tumor mutational burden
Ubl	ubiquitin-like
UCEC	uterine corpus endometrial carcinoma
UCS	uterine carcinosarcoma
UVM	uveal melanoma
WT	Wilms tumor
